# Submandibular gland-sparing radiation therapy for locally advanced oropharyngeal squamous cell carcinoma: patterns of failure and xerostomia outcomes

**DOI:** 10.1186/s13014-014-0255-x

**Published:** 2014-11-26

**Authors:** Michael F Gensheimer, Jay J Liao, Adam S Garden, George E Laramore, Upendra Parvathaneni

**Affiliations:** Department of Radiation Oncology, University of Washington Medical Center, Seattle, WA USA; Department of Radiation Oncology, The University of Texas MD Anderson Cancer Center, Houston, TX USA

**Keywords:** Head and neck cancer, Oropharynx, Xerostomia, Radiotherapy, Submandibular gland

## Abstract

**Background:**

Saliva from submandibular glands (SMG) is necessary to minimize xerostomia. It is unclear whether SMG can be safely spared in patients undergoing bilateral neck radiotherapy for locally advanced oropharyngeal cancer without increasing the risk of marginal recurrence. We evaluated the outcomes of contralateral submandibular gland (cSMG) sparing intensity-modulated radiation therapy (IMRT).

**Methods:**

All patients with stage III/IV oropharyngeal squamous cell carcinoma treated with bilateral neck IMRT from 2006–2012 at our institution were included. Appropriately selected patients with favorable primary tumor characteristics and no definite contralateral neck disease were treated with cSMG-sparing IMRT. Patterns of failure and xerostomia outcomes were retrospectively analyzed.

**Results:**

114 patients were treated. 89% had stage IV disease and 89% received definitive radiation therapy. 76 patients (67%) received cSMG sparing IMRT. With a median follow-up of 30 months, there were 10 local, 9 regional, and 10 distant recurrences. 2-year overall survival was 86% and 2-year loco-regional control was 87%. In cSMG spared patients, the mean cSMG dose was 30.7 Gy. Late grade 2+ xerostomia was significantly reduced in the cSMG spared group compared to those without SMG sparing (6 months: 23% vs. 72%, 12 months: 6% vs. 41%, 24 months: 3% vs. 36%, all p < 0.0007). There were no peri-SMG marginal recurrences in the cSMG-spared cohort.

**Conclusions:**

cSMG sparing IMRT did not increase marginal failures in this series of locally advanced oropharyngeal SCC patients. Xerostomia was significantly reduced in cSMG spared patients.

## Introduction

Oropharyngeal squamous cell cancer (OSCC) patients treated with radiotherapy (RT) are often cured of their disease [1]. However, chronic xerostomia remains a vexing and common clinical problem that impairs the quality of life of surviving patients. Sparing of salivary glands is essential to minimize xerostomia [1]. Parotid-sparing intensity-modulated radiation therapy (IMRT) is a standard radiotherapy technique for patients with squamous cancers of the head and neck region [2-6]. However, parotid saliva lacks mucins that maintain a patient’s subjective sense of hydration, and preserving the parotids alone has inconsistently translated to improvements in xerostomia [7-10]. Randomized studies comparing parotid-sparing IMRT with non-parotid-sparing techniques reported that despite better salivary flow rates with IMRT, the improvement in patient-reported xerostomia scores were modest [2-4]. One study demonstrated that the advantage of parotid sparing after therapy was <10 points on a 0–100 scale at one year, and regarded this difference as clinically insignificant [2]. Thus, parotid-sparing IMRT may be inadequate for maximizing patient-related xerostomia outcomes.

The submandibular glands (SMGs) contribute 65-90% of unstimulated saliva and account for almost 95% of salivary flow during a 24-hour period; this saliva is rich in mucins [7,8,11]. It has been reported that restricting the mean dose in one SMG to less than 39 Gy improved salivary flow and correlated with favorable rates of patient- and observer-rated xerostomia [12,13].

In patients treated with RT for a locally advanced oropharyngeal SCC, it is seldom possible to spare the ipsilateral SMG as it directly abuts the primary tumor and/or grossly involved lymph nodes that are treated to a tumoricidal dose of 66–70 Gy. However, it might be possible to spare the contralateral SMG (cSMG) since the level 1B nodes that lie anterior to the gland rarely harbor metastases from OSCC [14-17]. While it may be desirable to spare the cSMG, this is technically demanding due to its anatomical location in close proximity to levels II and III jugulodigastric nodal regions that are electively targeted during the treatment of advanced OSCC [8,14]. A primary OSCC that crosses the midline can also makes cSMG sparing difficult. Sparing of the cSMG is further hindered by the gland’s relatively small size. A treatment planning study of cSMG-sparing IMRT reported that in order to reduce the mean cSMG dose from 54 Gy to 40 Gy it was necessary to accept an under-dosage of target volumes in the vicinity of the gland to 90% of prescribed dose [18]. Due to these challenges, there are concerns that cSMG sparing IMRT could compromise dose to the adjacent target volumes in levels II and III and/or the primary tumor, leading to an increased risk of marginal recurrences [8,14].

There are no randomized trials evaluating the clinical outcomes of cSMG-sparing IMRT. Two small reports have compared the effectiveness of cSMG-sparing IMRT with non-SMG sparing IMRT [19,20]. While both groups found an improvement in xerostomia with cSMG sparing, the small sample sizes of 26 and 18 cSMG-spared patients make it difficult to rule out an increased marginal recurrence rate from cSMG sparing. Moreover, both series included patients with a variety of primary tumor sites, which could complicate their interpretation. Thus, it is unclear whether the cSMG could be spared to reduce long term xerostomia, without increasing the risk of a local-regional recurrence in patients with locally advanced oropharyngeal SCC requiring bilateral neck RT.

In mid-2006, at the University of Washington we commenced a policy of cSMG sparing IMRT, in addition to standard parotid sparing for the treatment of suitable OSCC patients. We report the outcomes in patients with locally advanced OSCC treated with bilateral neck IMRT at our institution. We divided the patients into cSMG-spared and cSMG-unspared groups in order to compare the incidence of peri-SMG marginal failures and xerostomia.

## Materials and methods

Institutional Review Board approval was obtained for this retrospective review of patients treated with IMRT for advanced OSCC.

### Inclusion/exclusion criteria

We identified all patients with stage III/IV OSCC treated with bilateral neck IMRT at our institution between 2006–2012. Patients treated for recurrent disease or early stage (stage I/II) disease, and those receiving unilateral treatment for well-lateralized tumors (e.g. early tonsil primaries) were excluded to minimize population heterogeneity. Six patients were excluded for short follow-up of <6 months.

For analysis of cSMG-sparing comparative outcomes, patients were divided into two groups, defined as follows: 1) cSMG spared: contralateral level IB nodal level was not targeted for elective RT and cSMG had an IMRT planning objective. 2) cSMG unspared: all other patients. Patients with nodal involvement limited to the unilateral neck and who had an anatomically favorable primary tumor were considered for cSMG sparing. Contraindications included a primary tumor extending close to the cSMG due to risk of under-dosing the tumor, or primary tumor involvement of the contralateral oral cavity, which would place the level IB space at risk.

### Staging and surveillance

Patients were staged per standard NCCN guidelines. PET or MRI scans were used as clinically indicated. Surveillance was every 3–4 monthly for 2–3 years, with increasing follow up intervals thereafter. Neck dissections were reserved for an incomplete response at >3 months or for progressive disease.

### Radiotherapy technique

**Figure 1:**
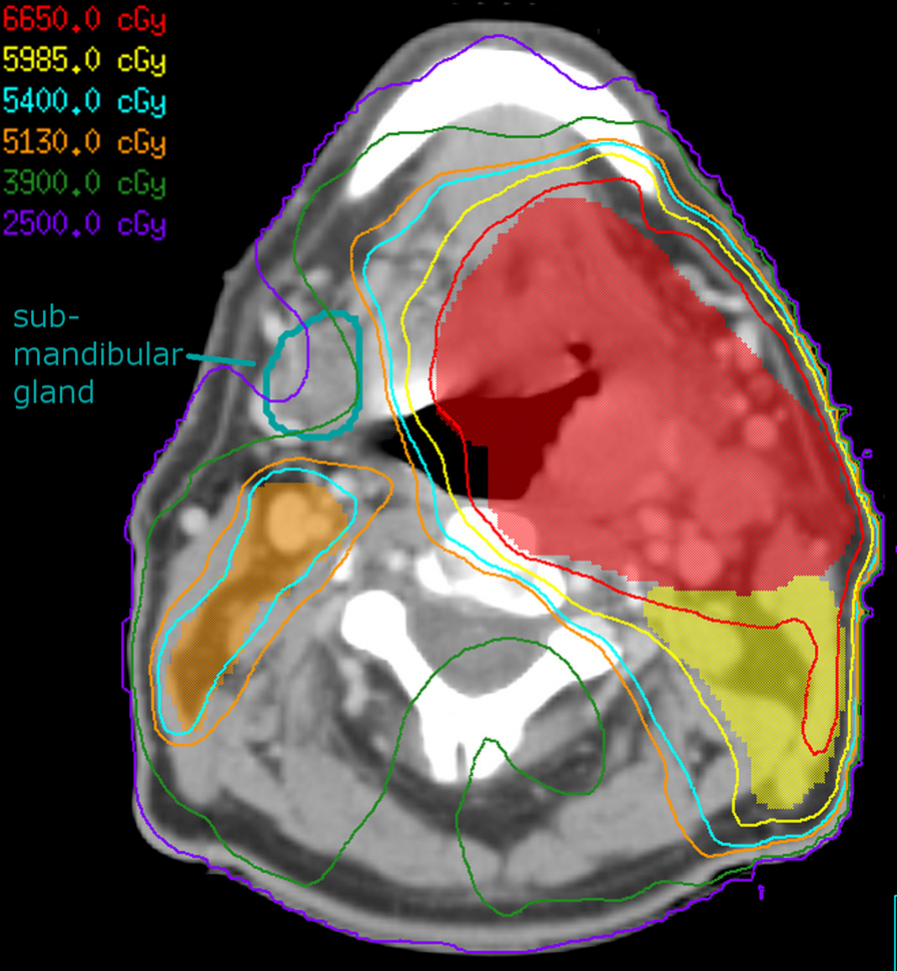
**Submandibular gland sparing treatment plan.** Treatment planning image for a T4N2b right oropharyngeal squamous cell carcinoma involving the lingual and pharyngeal tonsils, sparing the contralateral submandibular gland (cSMG). cSMG in teal. High-dose 70Gy PTV1 in red colorwash and corresponding 95% isodose line in red, 63Gy PTV2 in yellow colorwash and 95% isodose line in yellow, 57Gy PTV3 in orange colorwash and 95% isodose line in orange. Spared cSMG received a mean dose of 33Gy.

Unless there was low neck involvement, patients were treated with “split field” technique with multi-beam static IMRT to the primary and upper neck matched with a supraclavicular AP field using Elekta (Stockholm, Sweden) treatment machines and Pinnacle (Best, Netherlands) planning system. Nodal levels were defined per RTOG guidelines. A simultaneous integrated boost method was used. In patients with gross disease, the highest risk target volume (PTV1) was prescribed 70 Gy in 33 fractions; the intermediate and low risk subclinical volumes (PTV2 and PTV3) received 62.7 and 57 Gy, respectively. Two patients received a concomitant boost regimen. Patients treated postoperatively were prescribed 60, 57 and 54 Gy in 30 fractions to PTV1, PTV2, and PTV3. Regions harboring close or positive margins and/or extracapsular nodal spread were boosted to 63–66 Gy. In order to maximize salivary sparing, for PTV3, CTV to PTV expansion was limited to 2–5 mm. Although we covered level II adequately, the anterior extent of PTV3 contours were restricted to the great vessels (Figure 1). The contours of all cases were prospectively quality assured by 2 clinicians (UP & JJL), prior to dosimetric planning.

PTVs were covered by the 95% isodose line, although 90-95% coverage was accepted for PTV3 to optimize SMG sparing. SMG dose was pushed as low as possible without compromising coverage of PTV1 and PTV2. When possible, bilateral parotid glands were spared to achieve mean dose <24 Gy. Spinal cord with a 5 mm margin was limited to <45 Gy.

Patient positioning was verified with weekly MV port films or kV cone-beam CT. There was increasing utilization of daily cone-beam CT since its implementation in 2010.

### Statistical analysis

Kaplan-Meier overall survival, disease-free survival and loco-regional control were calculated. Event times for death/recurrence were calculated from the first day of RT. For disease-free survival calculations, events were recurrence and/or death. Viable tumor cells at time of salvage neck dissection were counted as a regional failure. Baseline characteristics were compared between the cSMG-spared and unspared groups using Fisher’s exact test and Wilcoxon rank sum test. Calculations were done with the statistical software R, version 3.0.2 (University of Auckland).

We recorded dosimetric parameters to determine target coverage. V93% (volume of target receiving at least 93% of prescribed dose) and D95% (minimum dose received by 95% of target volume) were recorded per RTOG 1216 [21]. Mean doses to cSMG and both parotids were recorded.

### Definition of “peri-cSMG” recurrence

In order to identify recurrences potentially attributable to SMG sparing, peri-cSMG recurrences were defined as any regional nodal relapse in levels I/II/III on the side of the cSMG, or primary tumor recurrence within 2 cm of the cSMG.

### Toxicity analysis

Late toxicity was retrospectively scored using the Common Terminology Criteria for Adverse Events version 4 [22]. Late toxicity was defined as occurring 6 months or more after the last day of RT. Grades of xerostomia were recorded. Factors predictive of late xerostomia were explored using univariate and multivariate logistic regression.

## Results

In total, 114 patients were treated. The patient characteristics are listed in Table 1. Seventy-six patients (67%) had cSMG sparing and 38 had non-cSMG-sparing IMRT. Eighty-nine percent of patients had stage IV disease. Eighty-nine percent of patients had definitive RT and 11% had post-operative adjuvant RT. Median highest prescribed dose was 70 Gy in 33 fractions (range 60–72 Gy). 89% received concurrent systemic therapy, most commonly cisplatin on a three weekly cycle. Fifteen percent of patients underwent consolidation neck dissection <6 months following definitive RT.

**Table 1:** **Patient characteristics**

	**cSMG spared (n = 76)**	**cSMG not spared (n = 38)**	**All patients (n = 114)**
Age at treatment			
Mean	56	56	56
Range	35-80	24-75	24-80
Radiation therapy role			
Definitive	65	37	102
Post-operative (oncologic primary resection)	11	1	12
Concurrent systemic therapy			
Yes	64	38	102
No	12	0	12
Disease subsite			
Tonsil	41	11	52
Base of tongue	32	20	52
Other/multiple	3	7	10
AJCC stage			
III	10	2	12
IVA	53	25	78
IVB	13	11	24
T stage			
1	14	2	16
2	28	5	33
3	15	13	28
4a	12	11	23
4b	7	7	14
N stage			
0	6	2	8
1	11	1	12
2a	11	1	12
2b	39	11	50
2c	3	17	20
3	6	6	12

**Figure 2:**
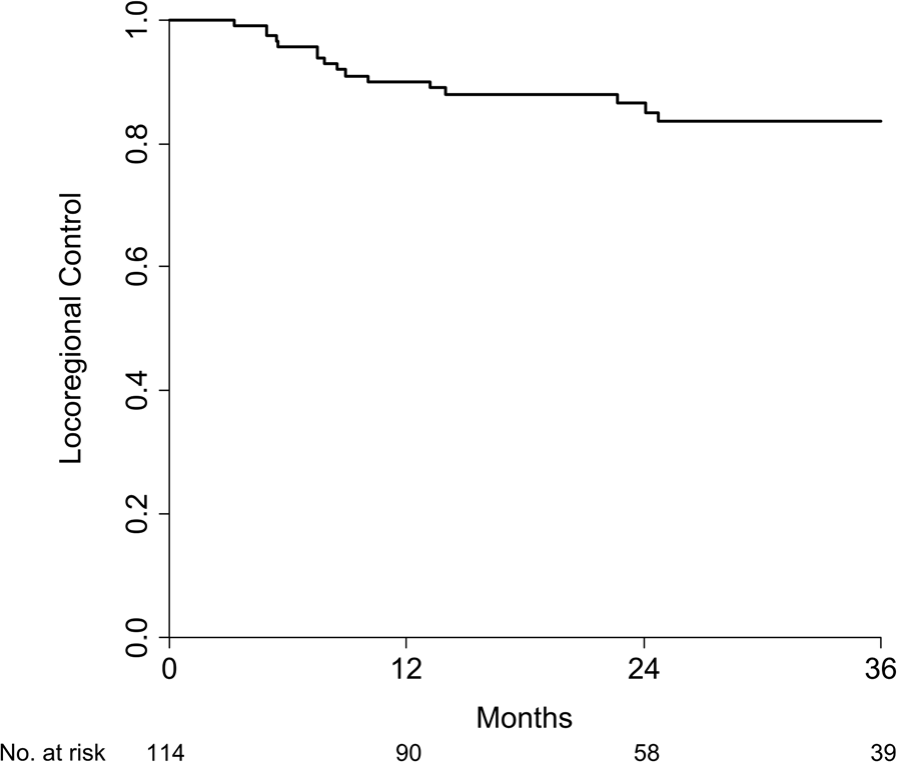
**Locoregional control in the entire group.** Kaplan-Meier-estimated locoregional control in patients with advanced oropharyngeal SCC treated with bilateral neck IMRT (n = 114).

Median follow-up in surviving patients was 30 months (range 8–86). Twenty patients have died. Twenty-three patients developed recurrent disease. Ten patients had disease recurrence in the primary site, nine in the regional nodes and ten in distant sites. Table 2 lists patterns of failure by treatment group. Two-year overall survival was 86%, 2-year disease-free survival was 77%, and 2-year locoregional control was 87% (Figure 2).

**Table 2:** **Patterns of failure**

	**cSMG spared (n = 76)**	**cSMG not spared (n = 38)**
Local recurrence	7	3
Regional recurrence	5	4
Distant recurrence	4	6
Any recurrence	13	10

Forty-eight patients were tested for HPV status based on either p16 immunohistochemistry or HPV DNA probe, and forty-two were positive. Of the sixty-six patients with unknown HPV/p16 status, thirty-five had fewer than 20 pack-years smoking history, which suggests that their cancers were likely to be HPV-associated [23].

### cSMG spared group: planning dosimetry and disease outcomes

Among the 76 patients who received cSMG sparing treatment, 87% had stage IV disease and 43% had T3/T4 tumors. 54% had tonsil and 42% had tongue base primaries. 86% had definitive RT.

The mean cSMG dose was 30.7 Gy (range 15.6-56.2). Mean cSMG dose <39 Gy and <50 Gy were achieved in sixty-two (82%) and seventy-two (95%) patients, respectively. The mean parotid dose on the SMG-spared side was 22.5 Gy (range 12.8-33.3).

Target PTV dosimetric coverage was not compromised by cSMG sparing. For PTV3 on the SMG-spared side, 72/76 of patients had V93% within the RTOG 1216 acceptable range of >/=97% of PD and 68/76 had D95% within the acceptable range of 95-107% of PD. Median V93% was 99% and median D95% was 100%.

At a median follow-up of 26 months, 64 patients are alive and 12 have died. Thirteen patients developed recurrent disease. Seven patients had disease recurrence in the primary site, five in the regional nodes and four in distant sites. Of the seven patients with primary site recurrences, none recurred within 2 cm of a spared SMG. Of the five patients with nodal recurrence, four had disease recurrence on the initial side of disease involvement. Only one patient had nodal disease recurrence on the side of a spared SMG, but disease recurred in level IV. Thus, there were no peri-SMG recurrences attributable to cSMG sparing IMRT. 2-year Kaplan-Meier overall survival was 88%; 2-year disease-free survival was 79% and 2-year locoregional control was 88%.

### cSMG unspared group

In thirty-eight patients, no attempt was made to spare the cSMG. Compared to the cSMG spared group, these patients were more likely to have T4 tumors (odds ratio 2.7, p = 0.02) and contralateral neck disease (odds ratio 18.8, p < 0.0001). While mean parotid gland dose was similar between the cSMG spared and unspared groups (32.1 vs. 34.4 Gy, p = 0.17), contralateral parotid dose was lower in the cSMG spared group (mean 22.5 vs. 27.8 Gy, p < 0.0001). We classified cSMG unspared patients into two groups indicating why the cSMG was not spared: 1) Twenty-six had no planning objective to spare the cSMG, due to contralateral neck disease (n = 21) or large primary tumors extending in close proximity to the cSMG (n = 5). 2) Twelve patients had elective targeting of the contralateral level IB nodal level due to significant extension of primary tumor into the oral tongue.

10/38 patients developed recurrent disease. Three patients had disease recurrence in the primary site, four in the regional nodes and six in distant sites. Of the 38 patients, only one had a recurrence near an unspared cSMG. This patient had a T4bN2c tumor and developed a contralateral level IIA recurrence centrally within the high dose volume. There was no planning objective to spare the cSMG in this case and the mean cSMG dose was 68 Gy. This patient also had synchronous distant metastases along with multiple sites of regional failure.

### Xerostomia outcomes

**Figure 3:**
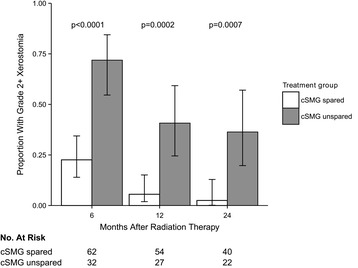
**Late grade 2+ xerostomia by treatment group.**

**Figure 4:**
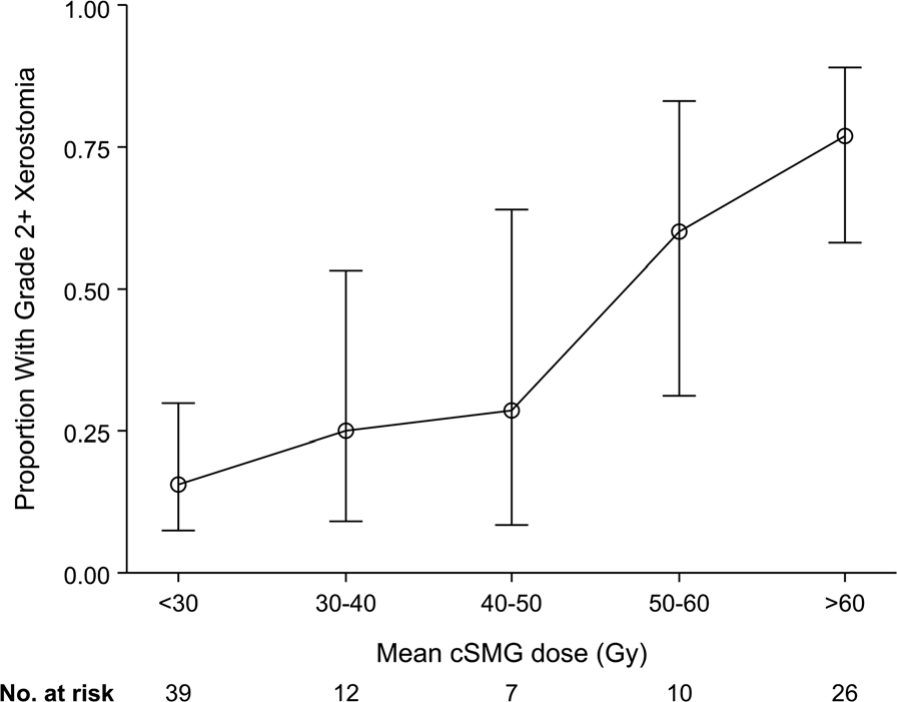
**Xerostomia at 6 months as a function of cSMG dose.** Error bars represent 95% confidence interval.

Xerostomia data at 6, 12, and 24 months after completion of RT were available for 94, 81, and 62 patients, respectively. Late grade 2+ xerostomia was significantly reduced in the cSMG spared group compared to those without SMG sparing (6 months: 23% vs. 72%, 12 months: 6% vs. 41%, 24 months: 3% vs. 36%, all p < 0.0007; Figure 3, Table 3). There was a positive correlation between cSMG mean dose and incidence of grade 2+ xerostomia at 6 months for the entire cohort of 114 patients (Figure 4). Because of the potential for confounders such as parotid gland dose, univariate and multivariate logistic regression predicting grade 2+ xerostomia at 6 months was performed (Table 4). Covariates were cSMG dose, contralateral parotid dose, T4 vs. T1-3 tumors, tongue base vs. other primary sites, bilateral vs. unilateral nodal disease, concurrent systemic therapy, and age at treatment. On univariate analysis, cSMG dose, contralateral parotid dose, T4 tumor, and presence of bilateral nodal disease were all positively correlated with xerostomia. On multivariate analysis, only cSMG dose (OR = 1.07 (1.02-1.11) per Gy, p = 0.002) and T4 tumor (OR = 3.90 (1.10-14.95), p = 0.04) were predictive of xerostomia. Mean bilateral parotid dose was analyzed as well and was not a significant predictor of xerostomia on univariate analysis (OR 1.03 (0.97-1.09), p = 0.31) or multivariate analysis (OR 0.99 (0.91-1.07), p = 0.75).

**Table 3:** **Prevalence of late xerostomia by treatment group**

	**cSMG spared**			**cSMG not spared**		
**Grade**	**6 months**	**12 months**	**24 months**	**6 months**	**12 months**	**24 months**
	**(n = 62)**	**(n = 54)**	**(n = 40)**	**(n = 32)**	**(n = 27)**	**(n = 22)**
0	11	12	16	1	5	5
1	37	39	23	8	11	9
2	12	3	1	21	11	8
3	2	0	0	2	0	0

**Table 4:** **Predictors of grade 2+ xerostomia at 6 months by univariate and multivariate logistic regression**

	**Univariate**		**Multivariate**	
**Variable**	**Odds ratio (95% CI)**	**P value**	**Odds ratio (95% CI)**	**P value**
Contralateral SMG dose (Gy)	1.07 (1.05-1.11)	<0.0001	1.07 (1.02-1.11)	0.002
Contralateral parotid dose (Gy)	1.10 (1.02-1.21)	0.03	0.92 (0.81-1.04)	0.19
T4 tumor	5.63 (2.21-15.31)	0.0004	3.90 (1.10-14.95)	0.04
Bilateral nodal disease	6.07 (2.26-17.85)	0.0005	2.67 (0.62-12.01)	0.19
Base of tongue primary	1.94 (0.84-4.55)	0.12	2.30 (0.71-7.95)	0.17
Concurrent systemic therapy	5.88 (1.01-111.52)	0.10	2.51 (0.33-53.76)	0.44
Age at treatment	1.03 (0.98-1.08)	0.31	1.02 (0.96-1.09)	0.56

## Discussion

While parotid-sparing IMRT improves salivary flow rates and is considered the standard technique for treating patients with locally advanced OSCC, it may be inadequate for patient-rated xerostomia, which has a significant adverse impact on quality of life [2,4,9,10]. To further diminish the negative consequences of xerostomia, in 2006 we began a program to spare the contralateral submandibular gland (cSMG) in patients with locally advanced oropharyngeal cancer without contralateral nodal involvement, and who had an anatomically suitable primary tumor. In 76 patients treated with cSMG sparing bilateral neck IMRT, we were able to limit the cSMG mean dose to 30.7 Gy. Xerostomia was significantly lower in the cSMG spared group compared to the unspared group, independent of parotid sparing. The magnitude of benefit from cSMG sparing seen in this study is notable. The PARSPORT randomized trial is the largest reported study comparing parotid sparing versus non-salivary sparing radiation therapy in oropharyngeal cancer patients [2]. In that study, parotid sparing resulted in a 25% absolute reduction in RTOG grade 2+ xerostomia at 12 months. By contrast, in the present study cSMG sparing resulted in a 35% reduction in grade 2+ xerostomia at 12 months. This benefit was durable, with reduction in xerostomia also seen at the 24 month time point. While these findings could be influenced by differences in patient characteristics between the cSMG-spared and unspared groups, the strong influence of cSMG dose on xerostomia persisted on multivariate analysis.

This is the largest report comparing outcomes from cSMG-sparing and non-cSMG sparing IMRT. Two smaller studies have been reported [19,20]. Saarilahti et al. reported on 18 patients treated with cSMG-sparing IMRT and compared their outcomes with 18 patients who did not have SMG sparing. Xerostomia was reduced in the SMG-spared group and there were no recurrences detected in the vicinity of the spared SMG. The SMG spared group had 8 patients with advanced stage OSCC. This series was subsequently updated with more patients, but without a comparative group [24]. Wang et al. reported that xerostomia at 6 months post-treatment was significantly improved among 26 patients treated with cSMG-sparing IMRT compared to 26 patients who received non-cSMG sparing IMRT. However, this difference did not persist at a longer follow up of 12 and 18 months [20]. There was one level II nodal failure in each group. Only five of their cSMG-spared patients had advanced stage OSCC. With the large sample size of >100 patients in the present study and the durable improvement seen in xerostomia, this report adds valuable information on the outcomes of cSMG-sparing IMRT in oropharynx cancer.

There are concerns that aggressive cSMG sparing could compromise adequate coverage of target volumes, with an increased risk of marginal recurrences [8,14]. Consequences of a marginal miss could be disastrous [25-28]. For example, peri-parotid recurrences have been observed in patients who had aggressive parotid sparing [25]. In order to maximize cSMG sparing, we included the great vessels of the neck (internal carotid and jugular) but not the region immediately posterior to the gland in the elective PTV (Figure 1). This represents a modification from the existing RTOG contouring guidelines [29]. Our definition of peri-SMG marginal recurrences was expected to be broad in identifying any failures that may have resulted as a consequence of SMG sparing. The lack of marginal relapses in the 76 cSMG spared patients with a median follow up of 26 months is reassuring. Since most recurrences in OSCC occur within 2–3 years after radiation therapy, the observed disease control rate should be maintained well over time.

There are other series reporting on the patterns of failure following cSMG-sparing IMRT for OSCC, although they did not have a cSMG unspared group to compare xerostomia outcomes (Table 5). The largest of these included 50 stage I-IV OSCC patients treated at Helsinki University Hospital along with 30 patients with other primary tumor locations. There were no recurrences in nodal levels I, II or the “vicinity” of the spared gland [24]. Other smaller series report similar outcomes [13,30,31]. Taken together, these experiences with over 150 patients demonstrate that cSMG sparing for oropharynx SCC patients can be achieved without increasing the risk of marginal failures. However, patient selection is crucial and we caution against sparing the cSMG when a large and infiltrative primary cancer extends in close proximity to the cSMG.

**Table 5:** **Literature on submandibular gland-sparing IMRT for oropharynx cancer**

**Study**	**N**	**Definitive RT**	**Mean cSMG dose (Gy)**	**Disease outcome**	**Late xerostomia**
Univ. of Washington (present report)	76	86%	30.7	No peri-SMG recurrence	23% grade 2+ at 6 months No permanent grade 3 +
Helsinki Univ., Finland [24]	50	49%*	27.8	No peri-SMG recurrence	No permanent grade 3+
VU Univ. Med. Ctr., The Netherlands [30]	20	100%	34.1	No peri-SMG nodal recurrence	Not reported
Univ. of Michigan [13]	17†	100%	~43	No contralateral level I recurrence	No grade 3+
Centre Eugene Marquis, France [31]	8	100%	33.8	No peri-SMG recurrence	No grade 3+

*Number is from larger series including other disease sites.

†Patients with contralateral SMG dose <50 Gy were extracted from a larger group of 78 patients (personal communication with Avraham Eisbruch). Of larger group, 92% had oropharynx cancer.

A limitation of our study is the retrospective, non-randomized design. There were known differences between the cSMG spared and unspared groups; for instance, there were more T4 tumors (47% vs 25%) in the unspared group. Although T4 stage was a significant predictor of worse xerostomia (p = 0.04), cSMG dose remained the strongest predictor (p = 0.002) in the multivariate analysis. We did not record objective salivary flow measurements or perform formal quality of life questionnaires. However, the observed clinical benefits of SMG sparing and the relationship between SMG dose and function in our series are consistent with the published literature [12,13,19,20].

## Conclusions

For patients with locally advanced OSCC, cSMG sparing is feasible in the majority of patients and may be safely attempted on the side of the neck being planned for elective nodal irradiation to reduce xerostomia and improve quality of life. The reduction in xerostomia in this series was large and clinically meaningful. In the absence of a randomized controlled study these observational data may be of value to clinicians considering cSMG-sparing IMRT for OSCC.
